# Long-term safety and tolerability of valsartan in children aged 6 to 17 years with hypertension

**DOI:** 10.1007/s00467-018-4114-0

**Published:** 2018-11-05

**Authors:** Randall Lou-Meda, Brigitte Stiller, Zenaida L. Antonio, Ewa Zielinska, Hui-Kim Yap, Hee Gyung Kang, Monique Tan, Robert D. Glazer, Michele A. Valentin, Linda Wang

**Affiliations:** 1 Fundación para el Niño Enfermo Renal/H. Roosevelt, 6 Avenida 9-18 zona 10 Edificio Sixtino II, Ala I, Oficina 804, Guatemala City, Guatemala; 2 University Heart Center Freiburg, Bad Krozingen, Department of Congenital Heart Fundación para el Niño Enfermo Renal Disease and Pediatric Cardiology, Medical Center and Faculty of Medicine, University of Freiburg, Freiburg im Breisgau, Germany; 3 Department of Pediatric Nephrology, National Kidney and Transplant Institute, Quezon City, Philippines; 4 Niepubliczny Zakład Opieki Zdrowotnej, Ezmed, Warsaw, Poland; 5 Department of Pediatrics, Yong Loo Lin School of Medicine, National University of Singapore, Singapore, Singapore; 6 Division of Pediatric Nephrology, Department of Pediatrics, Seoul National University Children’s Hospital, Seoul National University College of Medicine, Seoul, South Korea; 7 Novartis Pharmaceuticals Corporation, East Hanover, NJ USA; 8 Shanghai Novartis Trading Ltd, Shanghai, China

**Keywords:** Chronic kidney disease, Hypertension, Long-term safety, Paediatric, Valsartan

## Abstract

**Objective:**

The present study aimed to assess the long-term safety and tolerability of valsartan in hypertensive children aged 6–17 years, with or without chronic kidney disease (CKD).

**Methods:**

This was an 18-month, open-label, multicentre, prospective study conducted in 150 patients with history of hypertension with or without CKD. The primary endpoint was long-term safety and tolerability of valsartan and valsartan-based treatments, assessed in terms of adverse events (AEs), serious AEs, laboratory measurements, estimated glomerular filtration rate (eGFR), urinalysis and electrocardiogram.

**Results:**

Of 150 enrolled patients, 117 (78%) completed the study. At week 78, a clinically and statistically significant reduction in mean sitting systolic and diastolic blood pressures was observed in all patients (− 14.9 mmHg and − 10.6 mmHg, respectively). Within the first 3 months of treatment, mean urine albumin creatinine ratio decreased in CKD population, which was sustained. A higher percentage of CKD patients had at least one AE compared to non-CKD patients (85.3% vs. 73.3%, respectively). The majority of AEs were mild (50.7%) or moderate (18.7%) in severity. As expected, in patients with underlying CKD, increases in serum potassium, creatinine and blood urea nitrogen were more commonly reported compared to non-CKD patients. A > 25% decrease in Schwartz eGFR was observed in 28.4% of CKD patients and 13.5% of non-CKD patients.

**Conclusions:**

Valsartan was generally well tolerated, with an AE profile consistent with angiotensin receptor blockers in the overall population and in patients with underlying CKD. Long-term efficacy was maintained and a beneficial effect on proteinuria was observed.

## Introduction

Hypertension affects around one billion people globally, and it is estimated that this number will increase to 1.5 billion by 2025 [1]. In addition, hypertension is considered a major risk factor for cardiovascular disease, the leading cause of death worldwide [2]. Even though hypertension is more prevalent among adults in comparison to children [3], the prevalence of hypertension in the paediatric population is increasing and is estimated to be around 2–5% [4–8]. In most of the cases, hypertension in pre-pubertal children is secondary to an underlying disease (e.g. renal disease, coarctation of the aorta and endocrine disease), while essential hypertension is more common in adolescents and post-pubertal children [9–11]. The Fourth Report from the National High Blood Pressure Education Program (NHBPEP) Working Group on Children and Adolescents defines hypertension as an average systolic blood pressure (SBP) or diastolic blood pressure (DBP) that is ≥ 95th percentile for gender, age and height on at least three separate occasions. Average SBP or DBP levels that are ≥ 90th percentile but < 95th percentile, designated as ‘pre-hypertensive’, are considered an indication of heightened risk of developing hypertension [12].

There is a well-established relationship between the degree of hypertension and progressive kidney damage in children [13]. Data from the North American Pediatric Renal Trials and Collaborative Studies (NAPRTCS), 2006, showed that around 39.3% of children receiving antihypertensive treatment had chronic kidney disease (CKD) at the time of enrollment [14]. According to The National Kidney Foundation–Kidney Disease Outcomes Quality Initiative (NKF-KDOQI) guideline, classes of antihypertensive agents preferred for reducing cardiovascular risk and progression of CKD are angiotensin-converting enzyme inhibitors (ACEIs), angiotensin II receptor blockers (ARBs) and calcium channel blockers (CCBs) [15]. Among the preferred antihypertensive drugs, ARBs selectively inhibit the binding of angiotensin II to the angiotensin II type 1 receptor [16] and may also inhibit the action of angiotensin II synthesised by non-ACE-dependent pathways and control BP by differentially enhancing nitric oxide release [17]. ARBs help in reducing proteinuria in children and are particularly beneficial in BP management in children with proteinuria [18].

Valsartan, an angiotensin II receptor blocker, has shown to be efficacious and to provide dose-dependent reductions in BP [19–21]. In addition, valsartan has shown comparable efficacy to enalapril therapy in children with hypertension aged 6–17 years [21]. Although ARBs are commonly used in children, long-term safety and efficacy data for these antihypertensive drugs are scarce, particularly in children with CKD [22]. Hence, the current study focuses on evaluating the long-term safety and tolerability of valsartan, particularly in children with CKD.

## Patients and methods

### Study design

This was a prospective, open-label, multicentre (27 centres across 10 countries), 18-month study conducted from August 2011 to September 2015. This clinical study was designed, implemented and reported in accordance with the International Conference on Harmonisation (ICH) Harmonized Tripartite Guidelines for Good Clinical Practice, with applicable local regulations and the Declaration of Helsinki. The study was approved by local and central ethical review boards. Informed consent was obtained from a parent/guardian, and assent was obtained from the child, if applicable, before enrolling into the study. The study has been registered at ClinicalTrials.gov (identifier: NCT01365481) and on EudraCT (no: 2009-017594-37).

### Study participants

The study included male and female children aged 6 to 17 years, with or without CKD, body weight ≥ 18 kg and ≤ 160 kg and hypertension defined as mean sitting SBP (msSBP; mean of three measurements) that was ≥ 95th percentile and ≤ 25% above the 95th percentile for age, gender and height at baseline. At least 40% of enrolled patients were planned to be CKD patients. The original protocol defined CKD as patients with kidney damage for ≥ 3 months, as defined by estimated glomerular filtration rate (eGFR) of < 60 mL/min/1.73 m^2^ (by modified Schwartz formula) [23], or eGFR ≥ 60 and < 90 mL/min/1.73 m^2^ in addition to an abnormality in composition of urine, or in an imaging test or on kidney biopsy (i.e. patients with CKD stages 2 and 3). The CKD definition was subsequently amended to include patients with stage 1 CKD into the CKD cohort by removing the requirement that eGFR be < 90 mL/min/1.73 m^2^. This provided a broader range of CKD patients in the analysis population and is consistent with the CKD definition from the National Kidney Foundation (NKF) Kidney Disease Outcomes Quality Initiative (KDOQI) [24]. An independent adjudicator made final determination of the CKD status for patients originally classified as non-CKD. At baseline, patient demographics and other characteristics, including age, weight, msSBP, mean sitting DBP (msDBP), gender, race, CKD stage and concomitant antihypertensive medication, were obtained.

The key exclusion criteria were msSBP ≥ 25% above the 95th percentile for age, sex and height and presence of any clinically significant physical abnormality or clinically relevant abnormal laboratory values obtained at screening (other than those related to renal function), such as aspartate aminotransferase/serum glutamic oxaloacetic transaminase (AST/SGOT) or alanine aminotransferase/serum glutamic pyruvic transaminase (ALT/SGPT) > 3 times the upper limit of normal (ULN). Patients with active or chronic hepatitis, total bilirubin > 2 times ULN, eGFR < 30 mL/min/1.73 m^2^ (calculated using the modified Schwartz formula), white blood count < 3000/mm^3^, platelet count < 100,000/mm^3^, serum potassium > 5.3 mmol/L or haemoglobin < 8 g/dL were also excluded. Other key exclusion criteria were females of childbearing potential (unless willing to use highly effective contraception), pregnancy or lactation; uncontrolled diabetes mellitus as defined by the investigator; unilateral, bilateral and graft renal artery stenosis; current diagnosis of heart failure (New York Heart Association classes II–IV); or coarctation of the aorta.

Patients taking any of the following concomitant medications after screening, such as renin-angiotensin aldosterone system (RAAS) blockers other than study drug, lithium, potassium-sparing diuretics, potassium supplements, non-steroidal anti-inflammatory drugs (NSAIDS) and monoamine oxidase inhibitors, and patients with known or suspected contraindications to the study drug, including severe hepatic impairment, biliary cirrhosis, cholestasis and history of allergy to other ARBs or to ACEI and/or direct renin inhibitors, were also excluded.

### Study treatment

The study consisted of a screening phase before enrollment and washout and treatment phases. Patients previously taking RAAS blockers entered a washout period of 28 days (if msSBP increased above the 95th percentile for age, gender and height during the washout period, then patient stopped the washout and started with the study drug earlier). Background therapy of antihypertensive medication other than RAAS blockers (e.g. CCBs, diuretics and beta-blockers) were allowed from screening through the end of study. Patients taking non-RAAS blockers could also be tapered off these antihypertensives according to investigator instructions and manufacturer labelling in order to meet entry criteria.

**Fig. 1:**
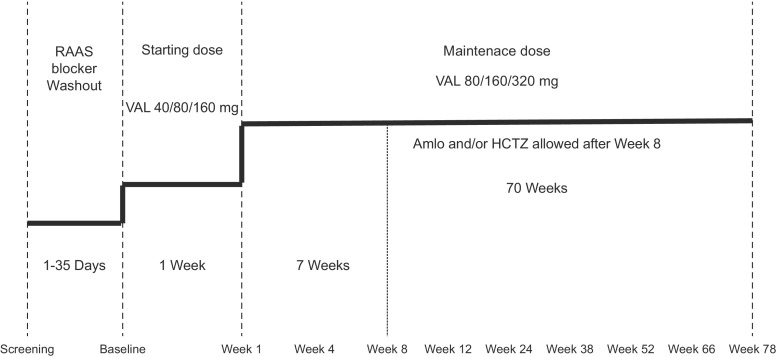
Study design. The starting and maintenance doses of valsartan were assigned according to three weight categories: ≥ 18 to < 35 kg, 40 mg and 80 mg; ≥ 35 to < 80 kg, 80 mg and 160 mg; and ≥ 80 to ≤ 160 kg, 160 mg and 320 mg, respectively. *Amlo* amlodipine, *HCTZ* hydrochlorothiazide, *RAAS* renin-angiotensin aldosterone system, *Val* valsartan

The treatment period began with a starting dose of valsartan of 40, 80 or 160 mg, depending on the patient’s body weight at baseline (Fig. 1). After 1 week, the dose was force titrated to 80, 160 or 320 mg, respectively. The starting and maintenance doses of valsartan were assigned according to three weight categories: ≥ 18 to < 35 kg, 40 mg and 80 mg; ≥ 35 to < 80 kg, 80 mg and 160 mg; and ≥ 80 to ≤ 160 kg, 160 mg and 320 mg, respectively. The study drug maintenance doses were maximum doses, and if required, they were down-titrated to the respective starting dose at the investigator’s discretion. After down-titration, the investigator may have also up-titrated the dose again to the maximum dose as necessary. At week 8 or later, if the msSBP and/or msDBP were higher than the 95th percentile for age, gender and height under the maintenance valsartan dose, then amlodipine and/or hydrochlorothiazide (dosage determined by the investigator) was added, or doses of existing background antihypertensive medication were adjusted at the discretion of the investigator. The overall treatment duration was 18 months.

### Study endpoints and assessments

#### Safety assessment

The primary endpoint of the study was to assess the long-term safety and tolerability of valsartan and valsartan-based treatments in children with hypertension, with or without CKD. Safety assessments were based mainly on the frequency of adverse events (AEs) and on the summary of laboratory measurements (haematology and biochemistry). Other safety data included vital signs, eGFR and electrocardiogram (ECG). All AEs and serious AEs (SAEs) with their severity and relationship to study drug were reported.

#### Efficacy assessments

The secondary endpoint of the study was to assess the long-term efficacy of valsartan and valsartan-based treatments in reducing and controlling msSBP and msDBP. Clinic BP was measured using a calibrated standard sphygmomanometer or a calibrated electronic (oscillometric) BP device with an appropriate cuff size (each site used their own device and the same staff member obtained BP measurements for the same patient, same time of day, using the same equipment at each visit). An average of three sitting BP measurements obtained at 2–3-min intervals was recorded as the msSBP and msDBP. The target mean BP was < 95th percentile for age, gender and height. The effect of valsartan and valsartan-based treatments on proteinuria in a subset of children with hypertension and CKD was also assessed. For CKD patients, the urine albumin creatinine (UACR) ratio was measured for urine specimens from three consecutive early morning urine collections, beginning 3 days prior to the scheduled visit.

### Statistical analysis

The patient demographic characteristics were summarised by treatment group. The full analysis set (FAS) included all patients who entered the treatment period, and safety set (SAF) included all patients who received at least one dose of the study medication. Summaries and analyses were presented for all patients and by patient groups per concomitant antihypertensive medication usage (with/without) and overall. The patient groups were determined by concomitant antihypertensive medication usage at any time during the treatment period, i.e. ‘valsartan alone’ and ‘valsartan antihypertensive’. Similar assessments were also made for the subgroups of CKD and non-CKD patients. For msSBP and msDBP, summary statistics for baseline, post-baseline and change from baseline were calculated by weeks and at endpoint (last observation carried forward [LOCF]) using the FAS. The number and percentage of patients achieving BP control (i.e. msSBP and msDBP < 95th percentile for age, gender and height) were calculated by visit and at endpoint (LOCF) using the FAS. Patients with a UACR reduction or increase of ≥ 50% from baseline and eGFR reduction ≥ 25% from baseline were summarised. The mean UACR was summarised by study week for CKD patients by concomitant antihypertensive medication (with/without) and overall for the SAF.

A simple paired *t* test for change from baseline was performed post hoc for msSBP, msDBP and mean UACR at each visit and also at the endpoint. In addition, comparison between CKD and non-CKD subgroups was performed using a one-way ANCOVA model for change from baseline in msSBP and msDBP at endpoint.

## Results

### Patient disposition

Of the 203 screened patients, 150 were enrolled and 117 (78.0%) completed the study. Out of the total 150 patients, 66.0% were males and a majority of the patients were from non-European countries (80.7%). All the patients were between 6 and 17 years of age; mean age, weight and body mass index were 13.36 years, 54.4 kg and 22.3 kg/m^2^, respectively.

Of the 150 patients, 41 were in the valsartan + antihypertensive group and 109 in the valsartan only group; 30 (73.2%) patients from the valsartan + antihypertensive group and 87 (79.8%) from the valsartan alone group completed the study. The majority of patients in the valsartan + antihypertensive group received dihydropyridine CCBs (68.3%) at some time during the study. The other commonly used antihypertensives were clonidine (14.6%) and thiazides (12.2%). Six (14.3%) patients in the valsartan + antihypertensive group received two other antihypertensives in addition to valsartan during the study.

Overall, there was a statistically significant difference in the racial distribution between the valsartan only and valsartan + antihypertensive groups. There was a higher percentage of Asians in the valsartan + antihypertensive group (51.2%) than in the valsartan only group (24.8%), and there was a higher percentage of Caucasians in the valsartan only group (47.7%) compared to the valsartan + antihypertensive group (14.6%).

Of the 150 patients, there were 75 CKD patients (of which 23 received valsartan + antihypertensives) and 75 non-CKD patients (of which 18 received valsartan + antihypertensives); of these, 53 (70.7%) CKD patients and 64 (85.3%) non-CKD patients completed the study. The most common primary aetiology of CKD was glomerulopathy [29 patients (38.7%)] followed by aplasia/hypoplasia/dysplasia [10 patients (13.3%)], reflux nephropathy [9 patients (12%)] and systemic (diabetes mellitus, systemic lupus erythematous) [9 patients (12%)]. The mean age for CKD patients was slightly lower than that for the non-CKD patients (12.49 years vs. 14.23 years, respectively). Majority of patients were from non-European countries; however, the percentage was higher in CKD patients compared to non-CKD patients (98.7% vs. 62.7%, respectively). Approximately half of the CKD patients (49.3%) were Asians compared to 14.7% of the non-CKD patients. The mean weight for CKD patients was lower than that for non-CKD patients (43.2 kg vs. 65.6 kg, respectively). The mean body mass index was also lower (20.3 kg/m^2^) for CKD patients compared to non-CKD patients (24.3 kg/m^2^). Patient demographics are presented in Table 1.

**Table 1:** Demographics and baseline characteristics by treatment and CKD status subgroups

Parameter	CKD			Non-CKD			Total		
	Valsartan + antihypertensive (*N* = 23)	Valsartan (*N* = 52)	Total (*N* = 75)	Valsartan + antihypertensive (*N* = 18)	Valsartan (*N* = 57)	Total (*N* = 75)	Valsartan + antihypertensives (*N* = 41)	Valsartan (*N* = 109)	Total (*N* = 150)
Age (years), mean (SD)	12.90 (3.35)	12.30 (3.20)	12.49 (3.23)	13.79 (2.64)	14.37 (2.83)	14.23 (2.78)	13.29 (3.05)	13.38 (3.17)	13.36 (3.13)
Age group (years), *n* (%)									
6–11	10 (43.5)	22 (42.3)	32 (42.7)	3 (16.7)	10 (17.5)	13 (17.3)	13 (31.7)	32 (29.4)	45 (30.0)
12–17	13 (56.5)	30 (57.7)	43 (57.3)	15 (83.3)	47 (82.5)	62 (82.7)	28 (68.3)	77 (70.6)	105 (70.0)
Gender, *n* (%)									
Male	11 (47.8)	36 (69.2)	47 (62.7)	15 (83.3)	37 (64.9)	52 (69.3)	26 (63.4)	73 (67.0)	99 (66.0)
Race, *n* (%)									
Caucasian	3 (13.0)	11 (21.2)	14 (18.7)	3 (16.7)	41 (71.9)	44 (58.7)	6 (14.6)	52 (47.7)	58 (38.7)
Asian	13 (56.5)	24 (46.2)	37 (49.3)	8 (44.4)	3 (5.3)	11 (14.7)	21 (51.2)	27 (24.8)	48 (32.0)
Native American	6 (26.1)	14 (26.9)	20 (26.7)	3 (16.7)	9 (15.8)	12 (16.0)	9 (22.0)	23 (21.1)	32 (21.3)
Other	1 (4.3)	3 (5.8)	4 (5.3)	4 (22.2)	4 (7.0)	8 (10.7)	5 (12.2)	7 (6.4)	12 (8.0)
Countries of distribution, *n* (%)									
Europe	1 (4.3)	0	1 (1.3)	3 (16.7)	25 (43.9)	28 (37.3)	4 (9.8)	25 (22.9)	29 (19.3)
Non-Europe	22 (95.7)	52 (100)	74 (98.7)	15 (83.3)	32 (56.1)	47 (62.7)	37 (90.2)	84 (77.1)	121 (80.7)
Weight (kg)									
Mean (SD)	44.8 (16.4)	42.5 (19.93)	43.2 (18.84)	59.8 (18.15)	67.4 (23.54)	65.6 (22.49)	51.4 (18.57)	55.6 (25.13)	54.4 (23.53)
Body mass index (kg/m^2^)									
Mean (SD)	21.1 (5.09)	19.9 (4.97)	20.3 (5.0)	22.9 (4.67)	24.7 (6.57)	24.3 (6.18)	21.9 (4.93)	22.4 (6.31)	22.3 (5.95)
Weight-adjusted dose (mg/kg)									
Mean (SD)	1.5 (0.33)	1.7 (0.34)	1.7 (0.35)	1.4 (0.28)	1.4 (0.32)	1.4 (0.31)	1.5 (0.31)	1.6 (0.36)	1.6 (0.34)
msSBP (mmHg)									
Mean (SD)	133.7 (11.26)	131.1 (13.07)	131.9 (12.52)	137.7 (10.97)	134.4 (10.5)	135.1 (10.63)	135.4 (11.17)	132.8 (11.85)	133.5 (11.69)
msDBP (mmHg)									
Mean (SD)	86.0 (13.70)	84.4 (11.03)	84.9 (11.84)	80.5 (11.58)	75.6 (9.27)	76.8 (10.02)	83.6 (12.95)	79.8 (11.03)	80.8 (11.66)
CKD, *n* (%)									
CKD	23 (100)	52 (100)	75 (100)	–	–	–	23 (56.1)	52 (47.7)	75 (50.0)
CKD stage 2 + stage 3	13 (56.5)	31 (59.6)	44 (58.7)	–	–	–	13 (31.7)	31 (28.4)	44 (29.3)
Non-CKD							18 (43.9)	57 (52.3)	75 (50.0)
Schwartz eGFR (mL/min/1.73 m^2^)									
Mean (SD)	97.2 (44.88)	100.7 (55.02)	99.7 (51.85)	152.4 (32.13)	158.5 (25.47)	157.0 (27.11)	121.4 (48.1)	130.9 (51.04)	128.3 (50.27)

The patient final CKD/non-CKD status information was from the adjudication form when available or from the investigator case report form when adjudication was not performed. CKD patients included all patients who were defined as stage 1, stage 2 and Stage 3 CKD patients

*CKD* chronic kidney disease, *eGFR* estimated glomerular filtration rate, *msDBP* mean sitting diastolic blood pressure, *msSBP* mean sitting systolic blood pressure

Overall, 33 (22%) patients discontinued the study: 11 (26.8%) in the valsartan + antihypertensive group and 22 (20.2%) in the valsartan only group. The main reasons for discontinuation were AEs, 17 (11.3%); lost to follow-up, 7 (4.7%); withdrew consent, 4 (2.7%); protocol deviations, 2 (1.3%); and discontinued due to abnormal laboratory values, unsatisfactory therapeutic effect and administrative problems, 1 (0.7%) patient each. Of the total CKD patient population, 22 (29.3%) discontinued the study; of these, 15 patients (20.0%) discontinued due to AEs.

At baseline, the msSBP was 133.5 mmHg and msDBP was 80.8 mmHg. The msSBP at baseline was slightly lower in CKD patients (131.9 mmHg) compared to non-CKD patients (135.1 mmHg). The msDBP for CKD patients (84.9 mmHg) was significantly higher than that for non-CKD patients (76.8 mmHg). Of the 150 patients enrolled, 106 (70.7%) patients had stage 1 hypertension and 41 (27.3%) had stage 2 hypertension. Two normotensive and one pre-hypertensive patient were randomised in error and were included in the analysis.

The mean Schwartz eGFR was 128.3 mL/min/1.73 m^2^, and a majority of the patients (76.0%) had eGFR ≥ 90 mL/min/1.73 m^2^. The mean Schwartz eGFR was lower in CKD patients (99.7 mL/min/1.73 m^2^) as compared to the non-CKD patients (157.0 mL/min/1.73 m^2^). Approximately half of the CKD patients (46.7%) and one non-CKD patient had eGFR < 90 mL/min/1.73 m^2^.

### Efficacy

#### Change in BP

**Fig. 2:**
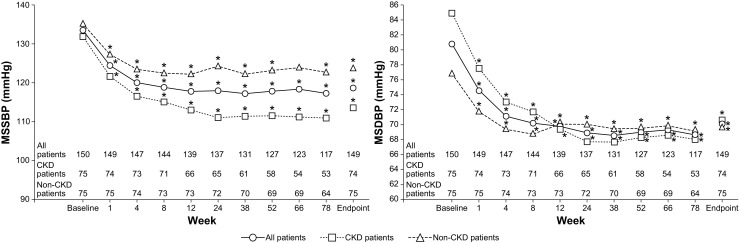
msSBP and msDBP in patients by visit (full analysis set). Number of patients at a given visit was the number of patients with both baseline and visit values at that visit. *p < 0.0001, baseline vs. post-baseline visit. *Antihyp* antihypertensives, *msDBP* mean sitting diastolic blood pressure, *msSBP* mean sitting systolic blood pressure

In the FAS, a clinically and statistically significant reduction in BP was observed from baseline to the endpoint (week 78 or LOCF) of treatment. There was a mean reduction in BP from 133.5 ± 11.69/80.8 ± 11.66 mmHg at baseline to 118.6 ± 14.81/70.1 ± 9.51 mmHg at the study endpoint (*p* < 0.0001). A clinically and statistically significant (*p* < 0.0001) reduction was observed as early as after 1 week of treatment (Fig. 2). In CKD patients, a mean reduction in BP was seen from 131.9 ± 12.52/84.9 ± 11.84 mmHg at baseline to 113.6 ± 14.15/70.6 ± 9.84 mmHg at the study endpoint (*p* < 0.0001) and in non-CKD patients, from 135.2 ± 10.64/76.8 ± 10.02 mmHg at baseline to 123.6 ± 13.79/69.7 ± 9.22 mmHg at the study endpoint (*p* < 0.0001).

**Fig. 3:**
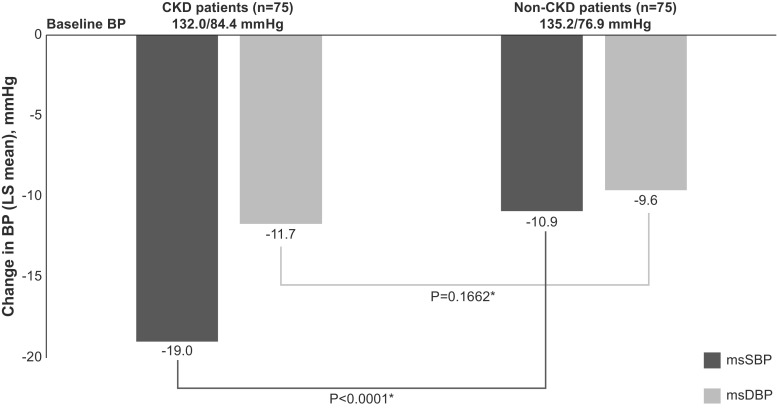
Change from baseline BP achieved in patients at the endpoint of treatment. The endpoint is week 78 or the last post-baseline observation carried forward value. *p values were based on an ANCOVA model with CKD strata as factors and centred-baseline MSBP/MSDP as the covariate. There was significant baseline imbalance (*p* < 0.05) in msDBP but not msSBP at baseline between CKD and non-CKD groups and a significant baseline-by-CKD-group interaction (*p* < 0.05) in assessment of difference between CKD and non-CKD groups in msDBP reduction from baseline. *BP* blood pressure, *CKD* chronic kidney disease, *LS *least square, *msDBP* mean sitting diastolic blood pressure, *msSBP* mean sitting systolic blood pressure

At the study endpoint, the least squares mean reductions from baseline in msSBP were 19.0 mmHg in CKD patients and 10.9 mmHg in non-CKD patients. The msSBP reduction in CKD patients was statistically significantly greater than in non-CKD patients (*p* < 0.0001) (Fig. 3). At the study endpoint, the mean reductions from baseline in msDBP were 11.7 mmHg in CKD patients and 9.6 mmHg in non-CKD patients (*p* = 0.1662 between subgroups). It should be noted that there was a significant imbalance in msDBP, but not msSBP, at baseline between the CKD and non-CKD subgroups and a significant baseline-by-CKD status interaction in msDBP reduction. Based on this significant baseline imbalance and interaction, no definitive conclusions can be made regarding comparisons between the CKD and non-CKD subgroups with respect to BP reductions.

#### BP control

Overall, 75.9% of all patients in the FAS had both msSBP and msDBP reduced to < 95th percentile for gender, age and height from ≥ 95th percentile at baseline. A total of 78.5 and 84.4% of patients from the FAS achieved msSBP and msDBP < 95th percentile for gender, age and height, respectively, at the study endpoint. The percentage of patients who achieved both msSBP and msDBP control was 83.8% in the valsartan alone group and 55.0% in the valsartan + antihypertensive group. The percentage of patients who achieved both msSBP and msDBP control to < 95th percentile for gender, age and height from ≥ 95th percentile at baseline was slightly higher in the CKD group (79.5%) compared to the non-CKD group (72.2%).

In a post hoc analysis, no statistically significant differences in predefined baseline characteristics were observed between patients who achieved blood pressure control and those who did not (data not shown).

### UACR

**Fig. 4:**
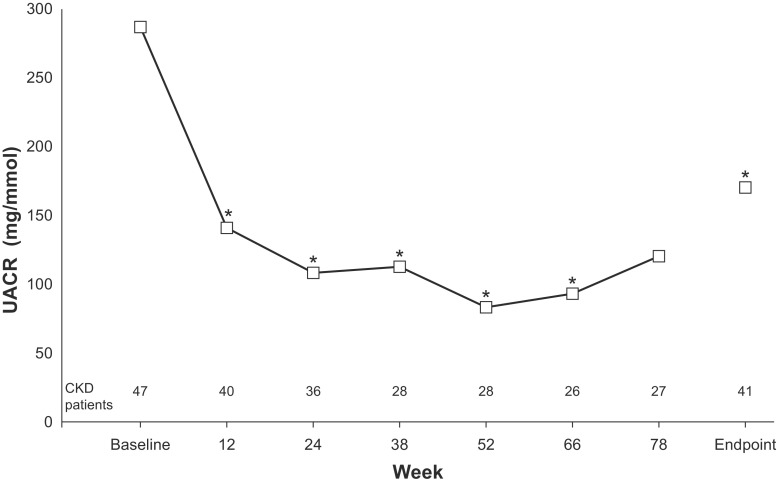
Mean UACR in CKD patients by visit. Number of patients at a given visit was the number of patients with both baseline and visit values at that visit. *p < 0.05. *Antihyp* antihypertensives,* UACR* urine albumin creatinine ratio, *VAL* valsartan

In the majority of patients, UACR either decreased or remained the same at the endpoint compared to baseline. UACR was reduced ≥ 50% from baseline in 43.9% of patients and remained the same (i.e. increase or reduction of less than 50% from baseline) in 41.5% of patients. Within the first 3 months, the mean UACR decreased in the CKD population (− 110 mg/mmol, *p* = 0.0019; *n* = 40 at week 12). At endpoint, the reduction was − 86 mg/mmol (*p* = 0.021; *n* = 41). The reduction was sustained for the duration of the study, but at the last visit (week 78), the mean reduction (− 98 mg/mmol; *n* = 27) did not reach statistical significance (*p* = 0.0634) (Fig. 4).

### Safety and tolerability

Valsartan was generally well tolerated in this long-term study. There were no reported deaths. The incidence of SAEs was higher in the valsartan + antihypertensive group (19.5%) compared to the valsartan alone group (6.4%). The occurrence of SAEs and discontinuations due to AEs were higher in patients with CKD compared to the overall population. Overall, 15 patients (10.0%) experienced a non-fatal SAE, of which 11 were CKD patients. Lupus nephritis (four patients, 2.7%) and pneumonia (two patients, 1.3%) were the most frequently reported SAEs. All other SAEs were reported in one patient only. All renal SAEs (lupus nephritis, CKD, proteinuria, decreased GFR, nephrotic syndrome, neurogenic bladder and immunoglobulin A (IgA) nephropathy) were reported by CKD patients and were not drug related.

Overall, 79.3% of patients reported at least one AE, with a higher incidence in the valsartan + antihypertensive group (90.2%) than in the valsartan alone group (75.2%). The majority of AEs were mild (50.7%) or moderate (18.7%) in severity. There were only 15 (10.0%) patients with severe AEs, of which 6 (14.6%) were in the valsartan + antihypertensive group and 9 (8.3%) were in the valsartan alone group. A higher percentage of CKD patients had at least one AE compared to non-CKD patients (85.3% vs. 73.3%, respectively). Cough, headache, pyrexia and nasopharyngitis were the most common AEs reported in the overall and CKD populations, with the incidence being slightly higher in CKD patients compared to non-CKD patients. Headache, dizziness and cough were the most common AEs in non-CKD patients (Tables 2 and 3).

**Table 2:** Number (%) of patients with AEs (≥ 5% in any group) in treatment period by preferred term (safety set)

	Valsartan + antihypertensives	Valsartan	Total
	*N* = 41	*N* = 109	*N* = 150
	*n* (%)	*n* (%)	*n* (%)
Any preferred term (total)	37 (90.2)	82 (75.2)	119 (79.3)
Headache	14 (34.1)	23 (21.1)	37 (24.7)
Cough	18 (43.9)	18 (16.5)	36 (24.0)
Nasopharyngitis	11 (26.8)	22 (20.2)	33 (22.0)
Pyrexia	12 (29.3)	18 (16.5)	30 (20.0)
Dizziness	8 (19.5)	17 (15.6)	25 (16.7)
Upper respiratory tract infection	2 (4.9)	17 (15.6)	19 (12.7)
Abdominal pain	4 (9.8)	7 (6.4)	11 (7.3)
Diarrhoea	2 (4.9)	8 (7.3)	10 (6.7)
Vomiting	5 (12.2)	4 (3.7)	9 (6.0)
Rhinorrhoea	4 (9.8)	3 (2.8)	7 (4.7)
Influenza	3 (7.3)	1 (0.9)	4 (2.7)
Lupus nephritis	3 (7.3)	1 (0.9)	4 (2.7)
Nasal congestion	3 (7.3)	1 (0.9)	4 (2.7)
Toothache	3 (7.3)	1 (0.9)	4 (2.7)
Pain in extremity	3 (7.3)	0	3 (2.0)

*AE* adverse event

**Table 3:** Number (%) of patients with AEs (≥ 5% in any group) in treatment period by CKD status by preferred term (safety set)

Preferred term	CKD			Non-CKD		
	Valsartan + antihypertensives	Valsartan	Total	Valsartan + antihypertensives	Valsartan	Total
	*N* = 23	*N* = 52	*N* = 75	*N* = 18	*N* = 57	*N* = 75
	*n* (%)	*n* (%)	*n* (%)	*n* (%)	*n* (%)	*n* (%)
Any preferred term (total)	21 (91.3)	43 (82.7)	64 (85.3)	16 (88.9)	39 (68.4)	55 (73.3)
Cough	12 (52.2)	12 (23.1)	24 (32.0)	6 (33.3)	6 (10.5)	12 (16.0)
Nasopharyngitis	7 (30.4)	14 (26.9)	21 (28.0)	4 (22.2)	8 (14.0)	12 (16.0)
Pyrexia	8 (34.8)	12 (23.1)	20 (26.7)	4 (22.2)	6 (10.5)	10 (13.3)
Headache	8 (34.8)	11 (21.2)	19 (25.3)	6 (33.3)	12 (21.1)	18 (24.0)
Upper respiratory tract infection	1 (4.3)	12 (23.1)	13 (17.3)	1 (5.6)	5 (8.8)	6 (8.0)
Dizziness	4 (17.4)	6 (11.5)	10 (13.3)	4 (22.2)	11 (19.3)	15 (20.0)
Vomiting	4 (17.4)	3 (5.8)	7 (9.3)	1 (5.6)	1 (1.8)	2 (2.7)
Abdominal pain	2 (8.7)	4 (7.7)	6 (8.0)	2 (11.1)	3 (5.3)	5 (6.7)
Diarrhoea	1 (4.3)	5 (9.6)	6 (8.0)	1 (5.6)	3 (5.3)	4 (5.3)
Hyperkalaemia	1 (4.3)	4 (7.7)	5 (6.7)	0	0	0
Rhinorrhoea	3 (13.0)	2 (3.8)	5 (6.7)	1 (5.6)	1 (1.8)	2 (2.7)
Urinary tract infection	2 (8.7)	3 (5.8)	5 (6.7)	0	1 (1.8)	1 (1.3)
Abdominal pain upper	2 (8.7)	2 (3.8)	4 (5.3)	0	3 (5.3)	3 (4.0)
Back pain	1 (4.3)	3 (5.8)	4 (5.3)	0	1 (1.8)	1 (1.3)
Chronic kidney disease	2 (8.7)	2 (3.8)	4 (5.3)	0	0	0
Hypotension	2 (8.7)	2 (3.8)	4 (5.3)	0	1 (1.8)	1 (1.3)
Lupus nephritis	3 (13.0)	1 (1.9)	4 (5.3)	0	0	0
Nausea	2 (8.7)	2 (3.8)	4 (5.3)	0	1 (1.8)	1 (1.3)
Respiratory tract infection	1 (4.3)	0	1 (1.3)	1 (5.6)	4 (7.0)	5 (6.7)

A patient with multiple adverse events for the same preferred term is counted only once

*AE* adverse event, *CKD* chronic kidney disease

The incidence of study drug-related AEs was lower in CKD patients compared to non-CKD patients (13.3% vs. 20.0%). Hyperkalaemia (4.0%) and hypotension (2.7%) were the most commonly reported drug-related AEs in CKD patients. Dizziness (10.7%) and headache (5.3%) were the most commonly reported drug-related AEs in non-CKD patients.

Seventeen patients discontinued from the study due to AEs: 15 (20%) were CKD patients and 2 (2.7%) were non-CKD patients. Seven patients discontinued from the study due to an AE, the AEs were considered suspected related to the study drug. The most frequent reasons for discontinuation in the CKD subgroup were decreased glomerular filtration rate in three patients (4.0%), chronic kidney disease in three patients (4.0%) and hyperkalaemia in two patients (2.7%). Two of the three patients discontinued for decreased GFR were CKD stage 3 at baseline and their final GFRs were 20 and 28 mL/min, respectively. The third patient was CKD stage 4 at baseline and was discontinued per protocol requirement at the following visit. Of the two patients discontinued for hyperkalaemia, one was CKD stage 1 and the other stage 3 at baseline. Serum potassium increased from 4.8 and 5.2 meq/L, respectively, at baseline to 5.3 and 5.4 meq/L, respectively, at final visit.

Patients with notable haematology abnormalities are presented in Table 4. The percentages of patients with the notable biochemistry abnormalities of an increase in serum potassium, creatinine and blood urea nitrogen and a decrease in eGFR at any time during the study were greater, as expected, in patients with underlying CKD compared to non-CKD patients (Table 5). No important differences were observed in other biochemistry tests. A > 25% decrease in Schwartz eGFR was observed in 10 (13.5%) of non-CKD patients and 21 (28.4%) of CKD patients. In approximately 50% of these CKD patients, the decrease was transient as a ≥ 25% decrease in GFR was observed in only 10 (13.9%) of CKD patients at study endpoint of which nine patients were in CKD stages 2 and 3.

**Table 4:** Number (%) of patients with clinically notable change in haematology values at any time during treatment period (safety set—CKD and non-CKD patients)

Laboratory test	Criterion	CKD patients	Non-CKD patients
		*N* = 75	*N* = 75
		*n*/*m* (%)	*n*/*m* (%)
Haematocrit	> 30% decrease	1/72 (1.4)	0/74
	> 50% increase	2/72 (2.8)	0/74
Haemoglobin	> 30% decrease	2/72 (2.8)	0/74
	> 50% increase	3/72 (4.2)	0/74
Platelet count (direct)	> 50% decrease	2/71 (2.8)	1/74 (1.4)
	> 75% increase	5/71 (7.0)	1/74 (1.4)
Red blood count	> 30% decrease	1/71 (1.4)	0/74
	> 50% increase	1/71 (1.4)	0/74
White blood count (total)	> 50% decrease	7/72 (9.7)	5/74 (6.8)
	> 50% increase	24/72 (33.3)	16/74 (21.6)

The % decrease/increase criterion is with respect to baseline. Values at ‘any time’ could come from post-baseline scheduled, unscheduled or premature discontinuation visits

*CKD* chronic kidney disease, *n* number of patients who met the specified criterion, *m* number of patients with both baseline and post-baseline values for the respective laboratory test

**Table 5:** Number (%) of patients with clinically notable change in key selected biochemistry values at any time during treatment period (safety population—CKD and non-CKD patients)

Laboratory test	Criterion	CKD patients	Non-CKD patients
		*N* = 75	*N* = 75
		*n*/*m* (%)	*n*/*m* (%)
Blood urea nitrogen	> 50% decrease	24/74 (32.4)	11/74 (14.9)
	> 100% increase	8/74 (10.8)	2/74 (2.7)
Creatinine	> 50% increase	13/74 (17.6)	4/74 (5.4)
	> 100% increase	3/74 (4.1)	0/74
eGFR	> 25% decrease	21/74 (28.4)	10/74 (13.5)
Potassium	> 20% decrease	4/74 (5.4)	7/74 (9.5)
	> 20% increase	26/74 (35.1)	11/74 (14.9)

The % decrease/increase criterion is with respect to baseline. Values at ‘any time’ could come from post-baseline scheduled, unscheduled or premature discontinuation visits

*CKD* chronic kidney disease, *eGFR* estimated glomerular filtration rate, *n* number of patients who met the specified criterion, *m* number of patients with both baseline and post-baseline values for the respective laboratory test

Evaluation of vital signs and ECG did not reveal any clinically relevant or unexpected adverse trends.

## Discussion

There are few clinical trials evaluating the treatment of hypertension in children, particularly those with CKD. The current study focused on the long-term safety and tolerability of valsartan along with the efficacy in paediatric patients aged 6 to 17 years with or without CKD. Most patients who enrolled in the present study also had a history of concurrent medical conditions other than hypertension. Approximately 50% had renal and/or urinary abnormalities, including congenital kidney disease. In the present study, clinically significant reductions in msSBP and msDBP with valsartan were noted in the valsartan alone and valsartan + antihypertensive groups. The results obtained in this study were consistent with a previous 3-month study in 261 children with hypertension aged 6–16 years, where valsartan effectively lowered SBP and DBP compared to enalapril [20]. The initial reduction in the msSBP and msDBP in the current study was clinically and statistically significant from week 1, and BP was well controlled in the long-term until endpoint (week 78 or LOCF) in both the overall population and the CKD and non-CKD subgroups. As noted previously, based on the significant imbalance in msDBP at baseline and the significant baseline-by-CKD status interaction in msDBP, no definitive conclusions can be made regarding comparisons between the CKD and non-CKD subgroups with respect to BP reductions.

Any comparisons between the valsartan alone and valsartan + antihypertensive groups should be avoided because these were not randomised treatment arms and any add-on antihypertensive medication was based on individual patients’ responses to valsartan treatment at various points in time and different treatment durations per individual patient situation.

Higher urine protein to creatinine ratio (UPCR) is an indicator of persistent proteinuria and an important risk factor for progressive renal failure and hyperfiltration injury [25]. UPCR levels decreased in a majority of CKD patients in a previously conducted 3-month study to a similar degree in patients receiving either valsartan or enalapril [20]. UACR levels (from morning urine collections) in this study either decreased or remained the same in a majority of the patients with CKD at the 78-week endpoint compared to baseline. The reduction in UACR was noted within 3 months after treatment, after which the reduction was sustained throughout the duration of the study in the overall CKD population. The reduction in UACR was statistically significant through week 66 and at study endpoint. The number of patients with UACR values at the final visit was reduced and despite a reduction in UACR, the *p* value approached, but did not reach statistical significance. Patients in the ESCAPE trial treated with the ACEI ramipril showed an initial decline in mean UPCR (86% of samples were 24-h urine samples) within the first 6 months of treatment. However, UPCR increased gradually to levels that were similar to the baseline over 3 years of continued ramipril treatment [26]. In both ESCAPE and the current study, there was a relatively high proportion of patients with missing values over the duration of treatment. It was speculated that the late increase in proteinuria in ESCAPE may be related to the ‘aldosterone breakthrough’ phenomenon with ACEIs thought to be related to upregulation of other enzymes like chymase. While this theoretically may be a reason for the difference in effect seen in our current study, there are studies where ‘aldosterone breakthrough’ has been reported with ARBs [27, 28]. In addition, different progressive courses of the underlying kidney disorders in the populations of ESCAPE and our current study may be postulated as an alternate explanation for the differences in courses in proteinuria. In this respect, these results must be interpreted with caution.

In the present long-term safety and tolerability study, valsartan was found to be safe and well tolerated. Most of the reported AEs were mild to moderate in intensity, and AEs that occurred in the study were not unexpected in the study population and/or are known to be associated with ARBs. Hyperkalaemia and hypotension were the most commonly reported drug-related AEs in CKD patients. The AE profile observed in the present study was generally similar to that reported in two previously conducted valsartan studies in children 6 to 17 years of age [19, 20].

Headache, cough, pyrexia and nasopharyngitis were the most common AEs reported in the overall and CKD population. In this study, headache was the most common AE reported in 24.7% of patients. This is consistent with the reported rates of headache in the general paediatric population where overall 17.1% of children aged 4 to 18 years were reported to have frequent or severe headaches, including migraine, in the previous 12 months with a higher prevalence with age [29]. The incidence of SAEs and discontinuations due to AEs were higher in CKD patients compared to the overall population. These SAEs and discontinuations due to AEs were mainly related to the underlying renal disease in these patients.

Increases in serum potassium, creatinine and blood urea nitrogen and a decrease in eGFR in patients with underlying CKD were observed. It is known that renin-angiotensin system inhibitors cause a decrease, often transient, in renal function as a result of decreased resistance at the efferent (post-glomerular) arteriole lowering intra-glomerular pressure and thus reducing GFR [30]. One also cannot exclude a normal progression of the underlying renal disease.

A limitation of the present study is that there was no control group and it was an open-label design. In addition, no direct formal comparison can be made in the difference in treatment response between the valsartan alone and the valsartan + antihypertensive subgroups or between the CKD and non-CKD subgroups since the groups were not prospectively randomised. These subgroups are considered to represent different populations and it was also noted that there was a difference in baseline blood pressures and a higher proportion of Asians in the CKD compared to the non-CKD subgroup. In addition, the patients in the valsartan + antihypertensive group received concomitant antihypertensive medication based on individual patients’ conditions at any time during the treatment period. Thus, no clear interpretation for potential treatment differences between subpopulations (e.g. CKD vs. non-CKD or ‘valsartan alone’ vs. ‘valsartan + antihypertensive’) can be made.

## Conclusions

In this population of hypertensive children aged 6 to 17 years with or without CKD, valsartan was generally well tolerated, with an AE profile consistent with that of an ARB in the overall population and in patients with underlying CKD. The efficacy of valsartan alone or in combination with other antihypertensives was maintained in the long term, and a beneficial effect on proteinuria was observed.
